# Resilience of hospital staff facing COVID-19 pandemic: Lessons from Israel

**DOI:** 10.3389/fpubh.2023.1050261

**Published:** 2023-03-31

**Authors:** Daniel Trotzky, Uri Aizik, Jonathan Mosery, Natali Carady, Guy Tavori, Aya Cohen, Gal Pachys, Miri Avraham, Osnat Levtzion-Korach, Orna Tal

**Affiliations:** ^1^Department of Emergency Medicine, Shamir Medical Center (Assaf Harofeh Medical Center), Zerifin, Israel; ^2^Sackler Faculty of Medicine, Tel Aviv University, Tel Aviv, Israel; ^3^Shamir Medical Center (Assaf Harofeh Medical Center), Zerifin, Israel; ^4^Faculty of Medicine, The Hebrew University of Jerusalem, Jerusalem, Israel; ^5^Nursing Administration, Shamir Medical Center (Assaf Harofeh Medical Center), Zerifin, Israel; ^6^Medical Management, Shamir Medical Center (Assaf Harofeh Medical Center), Zerifin, Israel; ^7^Israeli Center for Emerging Technologies, Zerifin, Israel; ^8^Department of Management, Bar Ilan University, Ramat Gan, Israel

**Keywords:** resilience, burnout, COVID-19 pandemic, healthcare workers, intensive care unit, emergency department, risk perception

## Abstract

**Introduction:**

The COVID-19 pandemic has placed additional burden on already strained healthcare systems worldwide, intensifying the responsibility and burden of healthcare workers. Although most hospital staff continued working during this stressful and challenging unprecedented pandemic, differences in the characteristics and attributes were noted between sectors and hospital departments. Israeli healthcare workers are trained and experienced in coping with national emergencies, but the pandemic has exposed variations in staff reactions. Understanding the intrinsic differences between sectors and departments is a key factor in staff and hospital preparedness for unexpected events, better resource utilization for timely interventions to mitigate risk and improve staff wellbeing.

**Objective:**

To identify and compare the level of resilience, secondary traumatization and burnout among hospital workers, between different sectors and hospital departments, during the COVID-19 pandemic.

**Methods:**

Cross-sectional research to assess the resiliency, secondary traumatization and burnout of healthcare workers at a large general public hospital in central Israel. The sample consisted of 655 participants across various hospital units exposed to COVID-19 patients.

**Results:**

Emergency department physicians had higher rates of resilience and lower rates of burnout and secondary traumatization than staff in other hospital departments. In contrast, staff from internal medicine departments demonstrated the highest levels of burnout (4.29). Overall, physicians demonstrated higher levels of resilience (7.26) and lower levels of burnout compared to other workers.

**Conclusion:**

Identifying resilience characteristics across hospital staff, sectors and departments can guide hospital management in education, preparation and training of healthcare workers for future large-scale health emergencies such as pandemics, natural disasters, and war.

## 1. Introduction

In early 2020, as confirmed cases of COVID-19 began to rise dramatically in countries across the world, the World Health Organization (WHO) declared COVID-19 a global pandemic. That declaration, along with uncertainty as to the virus characteristics and its threat, caused widespread concern. Within 2 years, the number of confirmed cases has reached over 507 million, and the death toll has topped nearly 6.2 million lives (1). The COVID-19 pandemic has caused a jump in mortality and morbidity, as well as a more subtle, yet equally damaging impact on the psychological status of not only its victims, but on healthcare workers (HCW) caring for the ill. The pandemic struck Israel's already overloaded health care system. Within 2 years, Israel had approximately 4 million confirmed cases, with 10,670 deaths (1). Although Israel's healthcare system has prepared for and been repeatedly tested during national emergencies, from earthquakes and mass-casualty events to military conflict, the pandemic posed a significant and unique challenge, especially to frontline HCW's. Frontline HCW's, primarily in emergency departments (ED) and intensive care units (ICU), managed fast-paced high-intensity work with the uncertainty and added stress of the pandemic.

The pandemic may have significantly eroded the resilience of both medical staff and healthcare systems due to infectious disease's inherent unpredictability, ability to impact even young, previously healthy patients and through instilling the fear of contracting the disease by the caregivers themselves (2). *Resilience* is defined as a person or organization's adaptation to stressful external sources such as trauma or threat (3). Resilience has been shown to play a beneficial role in reducing perceived workload and burnout. HCWs in dynamic, high-stress environment such as Hospitals, and especially in emergency departments (ED) and intensive care units (ICU), are particularly exposed by burnout (4). Resilience may differ between sectors of HCWs, such as between physicians and nurses, and vary based on personal and psychological characteristics (5).

Studies of infectious disease outbreaks have found a noticeable rise in the psychological distress, secondary traumatization and post-traumatic stress among HCW's (6). These variables may differ considerably among sectors of the population at large as well as sectors of HCW's. Frontline HCW's may be particularly vulnerable (7). Socio-demographic and professional characteristics, such as age, experience, education levels, and risk perception may alter the understanding and perception of the risk inherent in infectious disease outbreaks in the eyes of HCW's. Higher perceived risk has been demonstrated to positively correlate with increased psychological distress (8). It is critical to identify those sectors of HCW's with higher perceived risks of exposure to take steps to mitigate these concerns (9).

In previous pandemics, medical staff reported experiencing high levels of stress, anxiety, and depressive symptoms (10). Studies have reported adverse psychological reactions to the 2003 SARS outbreak (11), demonstrating that HCWs feared contagion and infection of their family, friends, and colleagues (12). Stressors grouped HCWs concerns into four categories: “fear of transmission”, “interference of workload with private life”, “uncertainty/lack of knowledge”, and “concerns about the team” (7). These acute concerns during the pandemic may be exacerbating already high rates of burnout amongst HCWs and leaving its mark on those that have cared for COVID-19 patients in the form of secondary traumatization.

*Secondary traumatization* is defined as “the stress deriving from helping others who are suffering or who have been traumatized” (13). As observed from previous viral outbreaks in recent times such as SARS, MERS, and Ebola, a large rise in secondary traumatization, psychological distress and post-traumatic stress in HCWs was reported during and following the pandemic. Secondary traumatization correlates with burnout (14).

In the UK, approximately 80% of physicians experienced burnout during the COVID-19 pandemic and reporting feelings of “psychological unease” (15). *Burnout* as determined by Freudenberger and Maslach (16) in the 1970's, can occur in any occupation, though higher levels of burnout have been reported among HCW's (17). Burnout is defined as failure or exhaustion because of excessive demands on energy, strength or resources in the workplace (18), thereby affecting patients' safety and health (19). Prior to the COVID pandemic, approximately half of physicians reported experiencing burnout (20). A 2018 Israeli Ministry of Health report identified the highest burnout among staff of ED and geriatric departments (21).

Burnout among HCW's could be negatively impacting the quality of patient care “in terms of adherence to guidelines, poor communication, medical errors, and patient outcomes and safety” (18). Burnout among staff may also contribute to patients' dissatisfaction with their treatment and an increase in complaints (22). In a large cross-sectional survey of HCWs in Taiwan in the first year of the COVID-19 pandemic, burnout was reported in 40.3% of respondents (22). The respondents who worked in the acute care division, compared with those who did not, had a 33.3% higher risk of burnout, especially, female, and therapists (physicians or nurses). A meta-analysis found that burnout was more prevalent in medical specialties with direct exposure to life-threatening situations, such as intensive care (18). Studies indicated that frontline HCWs may be at higher risk of negative emotional effects (23). In a study of ED physicians in Belgium, 1 in 3 met sub-clinical levels of anxiety and 14.5% met clinical levels for PTSD (24). Several studies in Spain have demonstrated that both female gender and less work experience among healthcare professionals correlated with increased susceptibility to the psychological effects of the pandemic (25, 26).

Remarkably, other studies have demonstrated the opposite; namely that frontline HCWs had lower levels of distress (27). A 2019 systemic review which analyzed findings from 31 research studies, found lower levels of secondary traumatization among first responders, possibly due to an immunization effect (14). Frontline HCWs may have been “immunized” or pre-conditioned for work during a pandemic by their previous experiences. A recent study demonstrated that oncologists who had been repositioned during the pandemic to work in frontline wards experienced lower levels of burnout than their colleagues, perhaps due to “immunization” from previous work experience (27).

There have been a limited number of studies that measured burnout among Israeli hospital workers. One study, conducted at a medical center in southern Israel demonstrated variability in burnout among different medical sectors during the first wave of the COVID-19 pandemic (28). A second study in Israel found that frontline HCW's including those in the ED and ICU were at higher risk of suffering from both stress and burnout (29).

Interestingly, both Israeli studies noted that HCW's were generally more concerned with the health of family members and friends than themselves. They also expressed concern and doubt for how the crisis was managed at the organizational and national levels.

The aim of our study was to determine levels of resilience, burnout and secondary traumatization in hospital departments and across sectors of HCWs during the second wave of the pandemic in Israel.

## 2. Materials and methods

### 2.1. Measurement tools to construct profile of HCW

#### 2.1.1. The Connor-Davidson resilience scale (CD-RISC-10)

We utilized the CD-RISC 10-itemScores in Non-treatment Seeking Trauma Survivors to measure four components of resilience: optimism, meaningfulness/purpose, resourcefulness/self-efficacy, and hardiness. Each question had five answer choices: not true at all (0), rarely true (1), sometimes true (2), usually true (3), mostly true (4), and true nearly all of the time (e.g., “always true”) (5). In addition, the total scoring scale was modified to be scored 1–10, with higher scores indicating higher resilience (30).

#### 2.1.2. Maslach burnout inventory (MBI)

An adoptive version containing 13 of the 16 components was utilized with a 10-point scale The higher the total score, the more severe the burnout (31).

#### 2.1.3. Professional quality of life scale (ProQOL)

Professional Quality of Life Scale (ProQOL) was utilized to measure secondary trauma, was with a 1 to 10 answer scale: 1 indicated that the participant never felt this emotion and 10 if the emotion was felt very often. Thus, the lower the ProQOL score, the lower the secondary trauma experienced. To assess secondary trauma, we used the validated official Hebrew ProQOL questionnaire 4th edition adapted modified version of 10 out of the 30 questions was used; those 10 questions focused on secondary traumatization (32).

### 2.2. Procedure

The research was presented to and approved by the hospital “Helsinki Committee” acting as the Independent Review Board (IRB) and Independent Ethics Committee (IEC). Consent was obtained in writing by participants, all of whom were adults. The study was conducted during the third wave of the pandemic in Israel, between February and March 2021, in a 900-bed hospital, Shamir Medical Center (SMC) (Assaf Harofeh). This hospital provides care for over one million residents of Israel's central region. SMC's ED is the 4th largest in Israel, treating about 160,000 patients each year. The questionnaire was electronically disseminated to all hospital workers following three reminders *via* SMS (phone text message) and to employees' organizational email. Study data were collected and managed using REDCap (Research Electronic Data Capture) electronic data capture tools hosted at SMC (33). REDCap is a secure, web-based software platform designed to support data capture for research studies (34).

### 2.3. Data analysis

Categorical variables are reported as frequency and percentage. Continuous variables are reported as mean and standard deviation. Continuous variables were evaluated for normal distribution using histograms and Q-Q plots. The Mann Whitney test and Kruskal Wallis tests were used to compare continuous variables, and Chi-square test was used to compare categorical variables. FDR (False Discovery Rate) was used to control for multiple comparisons. All statistical tests were two tailed, and a *P*-value of *P* < 0.05 was considered statistically significant. SPSS software was used for all statistical analysis (IBM SPSS statistic for windows, version 25, IBM corporation, Armond, New York, USA 2017). Regression models were used and presented separately in accompanying tables. Cronbach Alpha was utilized to gauge the internal consistency of our survey.

## 3. Results

The questionnaire was electronically disseminated to all 4,200 hospital workers (see Table 1). Participation rate among staff from the ICU was 55.7% (34 of 61 workers), among staff from internal medicine departments 22.7% (88 of 387 workers) and among ED staff 89.7% (166 of 185 workers). The overall participation rate among all hospital staff who completed the questionnaire was 15.6%.

**Table 1:** Distribution of resilience traumatization and burnout scores by population subgroups and characteristics (average, *P*-value).

**Category**	**Characteristics**	**Number of participants**	**Resilience (measured by CD)**	**Traumatization (measured by ProQOL)**	**Burnout (measured by MBI)**
	Total population	655	7.17	3.13	3.59
Gender	Men (31.3%)	205	7.46^**^	3.06	3.75
	Women (68.7%)	445	7.03^**^	3.16	3.51
Age	Below 40	273	7.20	3.10	3.85^*^
	Over 40	324	7.25	3.10	3.35^*^
Religion	Jewish	549	7.22	3.01^**^	3.45^##^
	Muslim	54	7.20	3.83^##^	4.09
	Christian	15	6.14^*^	3.79	4.94^*^
Profession	Physician	169	7.26^*^	3.22	3.61^*^
	Nurse	210	6.85^*^	3.36	4.12^**^
	Paramedic/PA	30	8.16^*^	2.94	3.27
	Hospital administration	139	7.47^*^	2.83^*^	2.99^*^
Department	Emergency department	166	7.41^**^	2.93	3.56
	Internal medicine department	88	6.93	3.85^*^	4.29^*^
	ICU	34	6.58^##^	3.44	3.94
Infected with COVID-19	Sick	79	6.81^##^	3.70^*^	3.72
	Not sick	568	7.21	3.04^*^	3.57
Vaccination status	Vaccinated twice	501	7.12	3.05	3.45

* *P* < 0.010;

** *P* < 0.020;

## *P* < 0.050.

We utilized Cronbach Alpha to gauge the internal consistency of our survey. To measure resilience, the survey consisted of 10 items and the value for Cronbach's Alpha was α = 0.910. For traumatization, the survey consisted of 10 items and the value for Cronbach's Alpha was α = 0.901. Finally, for burnout, the survey consisted of 13 items and the value for Cronbach's Alpha was α = 0.905 (Table 2).

**Table 2:** Cronbach alpha for resilience, traumatization, and burnout.

	**Cronbach's alpha**	**Number of items**
Resilience	0.910	10
Traumatization	0.901	10
Burnout	0.905	13

The average score for staff resilience was 7.17, men 7.46, females 7.03 (*P* = 0.020). Physicians' resilience score was 7.26, nurses 6.85 (*P* = 0.004), hospital administration workers 7.47 (*P* < 0.001), physician assistants and paramedics 8.16 (*P* < 0.001). The average resilience among ED staff was 7.41; among internal medicine staff 6.93 (*P* = 0.012), and ICU staff 6.58 (*P* = 0.041).

The average score for burnout was 3.59, with no significant difference between men and women (3.75 and 3.51, respectively). Physicians reported a burnout score of 3.61, nurses 4.12 (*P* = 0.009), hospital administration workers 2.99 (*P* < 0.001), and physician assistants and paramedics of 3.27 (*P* = 0.611). Distribution by departments revealed staff from internal medicine departments had burnout score of 4.29, ED and ICU staff scored 3.56 and 3.94, respectively (*P* < 0.001). Staff from internal medicine departments reported an average secondary traumatization score of 3.85 (*P* < 0.010), and ED staff reported an average score of 2.93. Physicians reported an average score of 3.22; nurses reported an average score of 3.36 (*P* = 0.608); and hospital administration staff reported an average traumatization score of 2.83 (*P* = 0.005). Regression models were used and presented in accompanying tables (see Table 3).

**Table 3:** Regression model—burnout.

**Dependent variable: Burnout**	**Unstandardized coefficients**		**Significance**	**95.0% confidence interval for B**	
	**B**	**Std. error**		**Lower bound**	**Upper bound**
Constant	3.904	0.367	0.000	3.183	4.624
Emergency department	0.005	0.190	0.980	−0.370	0.379
Internal medicine	0.721	0.249	0.004	0.230	1.211
Age	−0.017	0.007	0.011	−0.031	−0.004
Gender	0.394	0.176	0.025	0.049	0.739
Religion (Muslim, as compared to Jewish)	0.391	0.309	0.206	−0.216	0.999
Religion (Christian, as compared to Jewish)	1.252	0.492	0.011	0.285	2.218
Profession (Physician, Nurse, as compared to physician)	0.590	0.205	0.004	0.187	0.994
Profession (Paramedic/PA, as compared to physician)	−0.274	0.361	0.448	−0.983	0.435
Profession (Para-medical, as compared to physician)	−0.039	0.237	0.870	−0.504	0.427
Profession (Administration, as compared to physician)	−0.251	0.242	0.300	−0.728	0.225
Infected with COVID-19	0.341	0.487	0.484	−0.616	1.297
**Dependent variable: Resilience**	**Unstandardized coefficients**		**Significance**	**95.0% confidence interval for B**	
	**B**	**Std. error**		**Lower bound**	**Upper bound**
**Regression model—Resilience**					
Constant	7.079	0.336	0.000	6.417	7.740
Emergency department	0.228	0.176	0.197	−0.119	0.575
Internal medicine	−0.130	0.233	0.578	−0.589	0.329
Age	0.003	0.006	0.685	−0.010	0.015
Gender	0.214	0.162	0.187	−0.104	0.532
Religion (Muslim, as compared to Jewish)	−0.299	0.283	0.291	−0.854	0.257
Religion (Christian, as compared to Jewish)	−0.822	0.462	0.076	−1.729	0.086
Profession (Physician, Nurse, as compared to physician)	−0.128	0.191	0.505	−0.504	0.248
Profession (Paramedic/PA, as compared to physician)	0.647	0.338	0.056	−0.018	1.312
Profession (Para-medical, as compared to physician)	−0.410	0.220	0.063	−0.843	0.023
Profession (Administration, as compared to physician)	0.444	0.220	0.044	0.012	0.877
Infected with COVID-19	−0.491	0.457	0.283	−1.388	0.407
**Dependent variable: Traumatization**	**Unstandardized coefficients**		**Significance**	**95.0% confidence interval for B**	
	**B**	**Std. error**		**Lower bound**	**Upper bound**
**Regression model—Traumatization**					
Constant	2.476	0.360	0.000	1.769	3.183
Emergency department	−0.208	0.188	0.268	−0.577	0.161
Internal medicine	0.674	0.245	0.006	0.193	1.156
Age	0.011	0.007	0.109	−0.002	0.024
Gender	−0.090	0.173	0.601	−0.430	0.249
Religion (Muslim, as compared to Jewish)	1.167	0.297	0.000	0.583	1.750
Religion (Christian, as compared to Jewish)	0.801	0.484	0.099	−0.151	1.752
Profession (Physician, Nurse, as compared to physician)	0.176	0.201	0.382	−0.220	0.573
Profession (Paramedic/PA, as compared to physician)	0.226	0.355	0.524	−0.472	0.925
Profession (Para-medical, as compared to physician)	0.034	0.233	0.884	−0.424	0.491
Profession (Administration, as compared to physician)	−0.243	0.239	0.310	−0.713	0.227
Infected with COVID-19	−0.036	0.479	0.939	−0.978	0.905

## 4. Discussion

The levels of stress and burnout among hospital staff grew significantly during the COVID-19 pandemic worldwide (20). We noticed differences in resilience, burnout and secondary traumatization among hospital staff, and were intrigued to reveal the influential parameters that play a role in these differences. This study was aimed to highlight how and where hospital management can intervene in similar threats and crisis in the future.

Our findings reveal that ED staff showed higher resilience compared to colleagues in internal medicine wards, while staff from the ICU had the lowest resilience. This may be explained by a relative personal and professional scope, as ED staff members choose to work and are trained in front-line positions and pre-hospital care. Our primary assumption was that ICU staff members would have a similar level of resilience to ED staff, given the similar high-intensity work environment. However, a lower level of resilience in the ICU was observed. A possible explanation can be the unique circumstances in our hospital: first, during the pandemic ICU staff was assigned both to general ICU patients but at the same time to COVID-19 patients in special “COVID-19 ICU units” at two different sites on campus, bearing a larger burden. Second, ICU workers are exposed to much higher rates of patients' death in their routine practice compared to the ED, this was amplified during the pandemic, especially given the unexpected mortality rate in young, previously healthy patients. Third, senior workers from internal medicine departments were recruited to assist the ICU team, generating a subset of workers that were “new to the job” and unfamiliar with their colleagues, resulting in a low cohesive team that expressed lower support in peers.

Indeed, following our policy to recruit internal medicine staff to assist specially designated COVID-19 wards, we observed that while these staff members were separated from their original department colleagues, it dramatically impaired their social supportive network, beyond placing additional strains on the remained staff in internal medicine wards. Previous studies have highlighted the importance of workplace and social support systems as a leading factor in combating workplace stressors (7). During the first and second waves of the pandemic, the combination of reduced organic sustenance from familiar colleagues, and the added trauma of being involved in the care of numerous critically ill patients, was remarkable; we witnessed burnout and secondary traumatization rates significantly increased specifically in internal medicine departments. In contrast, hospital administration workers had the lowest rate of secondary traumatization, probably due to minimal patient contact.

Other indirect effects of the pandemic were that internal medicine departments had a much greater patients load during the pandemic, in addition to the fact that internal medicine departments in Israel are constantly understaffed and overloaded with complex patients. The guidelines and instructions regarding COVID-19 patients also changed as awareness and knowledge of the virus grew, and protocols evolved, augmenting complexity and stress to staff workload. The flood of studies, reports and medical advice in medical journals and public resources complicated efforts to establish precise effective protocols for staff on the wards. This lack of clarity in protocols for dealing with COVID-19 patients, increasing uncertainty and stress (11).

The rates of COVID-19 infection were significantly lower among ICU staff, not only due to exposure to a smaller load of patients, but also due to increased personal protective equipment availability and use, as well as awareness to the most sever situation of the disease, similar to the literature (35). Resilience after being personally infected by the virus was lower however those being vaccinated showed higher resilience. We may carefully assume fear of death played a role in workers that personally experienced the disease.

Though the rate of illness in the ED workers was the highest, they still maintained the uppermost resilient population. We believe this is due to the nature of work in the ED that may pre-condition staff to stressful, and the high cohesiveness of ED teams within the demanding environment, both during routine and extraordinary circumstances.

Resilience significant differed between sectors: physicians had a higher resilience compared to nurses and hospital administration workers. We believe that is due to the physicians' general medical knowledge, education, training for swift respond to emergencies and a better understanding of the emerging threat of a global pandemic. Others suggest physicians also maintain a more pronounced internal locus of control increasing confidence (18).

Burnout was reported lower by physicians compared to nurses. This can be explained as nurses spend significantly more time in direct contact and care of COVID-19 patients. Moreover, in our study nurses were more affected by COVID-19 themselves. A former study confirms that “being a nurse and female conferred great risk of acquiring trauma or stress-related disorders, depression and anxiety” (36). We found men were more resilient than women, yet we assume this may be correlated to the fact that the majority of nurses in our hospital are women. Others have pointed to “background stressors” to account for gender differences in burnout and mental stress, which includes caring for children and other dependents during the pandemic (37).

Other sectors of HCWs including physician assistants, paramedics, and administrators reported lower levels of burnout. Studies have reported that junior hospital staff generally exhibited milder stress symptoms including anxiety, depression, and insomnia compared to more experienced workers (11). This can be explained by a lower risk perception in younger staff members either to the virus, or transmitting it to friends and family, meaning a lower accountability sense. In contrast, older workers may see themselves more vulnerable due to high age, chronic medical conditions, fear from complications, or a personal elevated risk perception based on their own experience.

Finally, while analyzing socio-demographic subpopulation, we observed that males of the majority subgroup had significantly lower burnout and higher resilience compared to the minority subgroup. This can be explained by the unique feature of Israeli lifestyle: exposure to terror, the mandatory military service of Jewish men and women, as well as cultural behavioral characteristics. We assume military training and past combat experience, and high alert to terror events, may prepare our medical workers for physical, psychological and emotional challenges, their resilience and lowering traumatization.

## 5. Conclusion

Though Israel is accustomed to the dynamic and stressful nature inherent in national emergency situations, significant differences in responses and attitudes were detected to the COVID-19 pandemic between departments and sectors in our hospital. This was unexpected, as many Israeli HCWs have previously served in military positions themselves under Israel's mandatory military service law, thereby providing additional training and mental hardiness; essential tools in national health crises to both reduce burnout and secondary traumatization as well as improve performance.

The focused findings can be used as a platform for hospital leadership and department directors in improving staff training, preparation and acceptance in future events, and contribute to building capable effective health force, improving staff emotional health, motivation and commitment. This can also provide an opportunity to maximize staff utilization for improved overall hospital performance. The parameters who fit the profile of “resilient HCW in hazards events” may be anchors for other workers, especially young and unexperienced, during future global health crises to improve clinical patient outcomes and HCW wellbeing. Healthcare leadership can design and initiate support programs for those sectors of HCWs who have been highlighted to be most “at risk” during such challenging times. Additional longitudinal research is needed to assess resilience over time.

## 6. Limitations

This study has several limitations. Firstly, a small sample of medical staff from a single hospital answered the survey. It is important to note however, that the hospital where this study took place is, for the purpose of this study, a microcosm of the diverse country at large. The heterogeneous workforce includes representatives of Israel's large religious groups, including Jews, Muslims, Christians and Druze, as well as multiple nationalities in similar percentages as those groups are represented in the country. As such, this study has a higher external validity than what would otherwise be expected.

As was mentioned in the study, Israel has a mandatory military service, unlike most other large Western democracies. This variable may have impacted the outcome of this study, specifically traumatization and resilience. Many studies have highlighted the effects of living in Israel with trauma and resilience (38).

Finally, the study also had a lower-than-expected participation rate in spite of our efforts to recruit higher numbers.

## Data availability statement

The datasets presented in this article are not readily available because data can be made available upon request to the corresponding author and in accordance with Israeli public health and privacy laws. Requests to access the datasets should be directed to AC, ayaco@shamir.gov.il.

## Ethics statement

The studies involving human participants were reviewed and approved by Shamir Medical Center's Helsinki Committee. Written informed consent for participation was not required for this study in accordance with the national legislation and the institutional requirements.

## Author contributions

DT was involved with conceptualization, data curation, formal analysis, investigation, methodology, supervision, visualization, drafting the manuscript, and reviewing and editing the manuscript. UA was involved with conceptualization, data curation, formal analysis, investigation, methodology, project administration, visualization, drafting the manuscript, and reviewing and editing the manuscript. JM was involved with drafting the manuscript and reviewing and editing the manuscript. NC was involved with data curation and reviewing and editing the manuscript. GT was involved with conceptualization, methodology, and reviewing and editing the manuscript. AC was involved with project administration. GP was involved with visualization and reviewing and editing the manuscript. MA and OL-K was involved with supervision, visualization, and reviewing and editing the manuscript. OT was involved with supervision, visualization, drafting the manuscript, and reviewing and editing the manuscript. All authors contributed to the article and approved the submitted version.
